# Investigation of the Interaction between Human Serum Albumin and Branched Short-Chain Perfluoroalkyl Compounds

**DOI:** 10.1021/acs.chemrestox.2c00211

**Published:** 2022-09-23

**Authors:** Giulia Moro, Stefano Liberi, Filippo Vascon, Sara Linciano, Sofia De Felice, Silvano Fasolato, Carlo Foresta, Luca De Toni, Andrea Di Nisio, Laura Cendron, Alessandro Angelini

**Affiliations:** † Department of Molecular Sciences and Nanosystems, Ca’ Foscari University of Venice, Via Torino 155, 30172 Venice, Italy; ‡ Department of Biology, University of Padua, Viale G. Colombo 3, 35131 Padua, Italy; § Department of Medicine, University of Padua, Via Giustiniani 2, 35128 Padua, Italy; ∥ Department of Medicine, Unit of Andrology and Reproductive Medicine, University of Padua, Via Giustiniani 2, 35128 Padua, Italy; ⊥ European Centre for Living Technology (ECLT), Ca’ Bottacin, Dorsoduro 3911, Calle Crosera, 30123 Venice, Italy

## Abstract

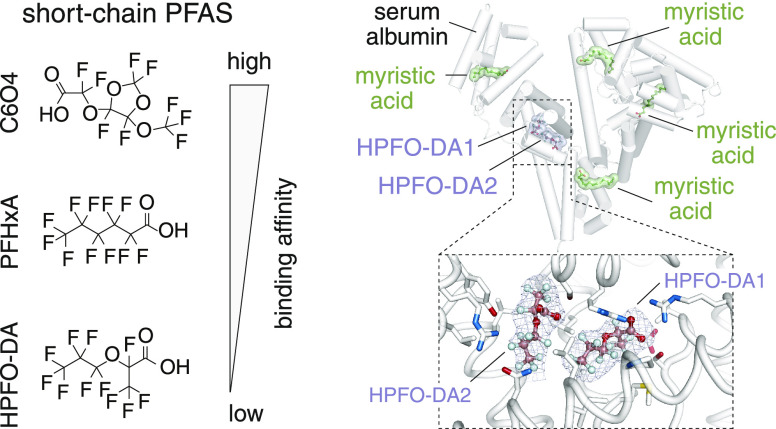

The current trend dealing with the production of per- and polyfluoroalkyl substances (PFASs) involves the shifting toward branched short-chain fluorinated compounds known as new-generation PFASs. A key aspect to be clarified, to address the adverse health effects associated with the exposure to PFASs, is their binding mode to human serum albumin (hSA), the most abundant protein in plasma. In this study, we investigated the interaction between hSA and two representative branched short-chain PFASs, namely, HPFO-DA and C6O4. In-solution studies revealed that both compounds bind hSA with affinities and stoichiometries lower than that of the legacy long-chain perfluoroalkyl compound PFOA. Competition experiments using hSA-binding drugs with known site-selectivity revealed that both HPFO-DA and C6O4 bound to pockets located in subdomain IIIA. The crystal structure of hSA in complex with HPFO-DA unveiled the presence of two binding sites. The characterization and direct comparison of hSA interactions with new-generation PFASs may be key elements for the understanding of the toxicological impact of these compounds.

## 1 Introduction

Per- and polyfluoroalkyl substances (PFASs) represent a large class of synthetic compounds that comprise a linear or branched carbon chain.^1−4^ PFASs are divided into long- and short-chain compounds based on the length of their fully or partially fluorinated carbon chain.^5,6^ Long-chain PFASs possess a six or more-perfluorinated carbon backbone, while short-chain ones have less than six perfluorinated carbons.^5,6^ The most common long-chain PFASs are perfluorooctanoic acid (PFOA) and perfluorooctanesulfonic acid (PFOS).^6−8^ Both PFOA and PFOS are characterized by a high chemical stability, accounting for their widespread use in manufacturing and consumer goods.^9−11^ However, the high resistance to degradation allowed them to persist in multiple environmental matrices and bioaccumulate within living organisms with harmful consequences to animal and human health.^12−15^ This evidence has ultimately prompted the competent authorities to implement actions aimed at reducing long-chain PFAS production and emissions.^7,16−18^

In response to the strict regulation limits and the phase out actions, short-chain alternatives represent the most attractive targets, from an industry environmental perspective, compared to long-chain PFASs.^19−23^ Based on their chemical structure, these alternative fluorinated compounds can be divided into two main categories: perfluoropolyethers with different functional head groups (mainly carboxylic and sulfonic ones) and shorter-chain homologues of long-chain PFASs.^20,24−26^ Common short-chain perfluoropolyether replacements are 2,3,3,3-tetrafluoro-2-(heptafluoropropoxy) propanoic acid (HPFO-DA), also known as GenX,^27^ and perfluoro ([5-methoxy-1,3-dioxolan-4-yl]oxy) acetic acid (C6O4).^28^ HPFO-DA was initially introduced as a safer alternative of PFOA and widely applied in manufacturing.^29^ Since its introduction, the environmental impact of HPFO-DA was carefully monitored and its adverse effects on human health found to be similar to those of PFOA and PFOS.^26,30^ These findings led the U.S. Environmental Protection Agency and other competent authorities to develop large-scale monitoring plans and declare HPFO-DA a potential carcinogenic agent.^17,31^ Oppositely, less information is available regarding C6O4, an alternative to long-chain PFASs, which was considered safe for use in food contact materials by the European Food Safety Agency,^32^ and has shown less toxicity in various cell models in vitro.^33,34^

Major health effects of long-chain PFASs are associated with their low elimination rates and high accumulation levels in the blood and in vital organs.^35^ Indeed, the average half-life values for serum elimination of legacy PFASs, such as PFOA and PFOS, in environmentally exposed human populations are estimated to be in the order of 1–5 years, depending on the type of compound.^36,37^ Such long persistence and accumulation of long-chain PFASs in circulation have been ascribed to their ability to bind human serum albumin (hSA), the most abundant protein in plasma with a maximum circulatory half-life of 19 days in humans.^38,39^ hSA can bind a large diversity of PFASs, shielding their hydrophobic character and strongly enhancing their absorption and distribution throughout the body ultimately leading to relatively high blood concentration.^40^ While considerable insights into the binding mode of several long-chain PFASs with hSA have been described over the last years,^40−45^ a detailed analysis of the interaction of new-generation PFASs with hSA has not been reported yet.

In the present study, we applied isothermal titration calorimetry (ITC) and X-ray crystallography to characterize the interaction of hSA with two representative branched short-chain perfluoropolyethers, namely HPFO-DA and C6O4. Competition experiments with known hSA-binding drugs revealed that both compounds bound to pockets located in a single subdomain. The crystal structure of hSA in complex with HPFO-DA unveiled the presence of two binding sites. The elucidation of the molecular basis of the interaction between hSA and short-chain PFAS alternatives is expected to provide a better assessment of the absorption and elimination processes of these toxic compounds in vivo.

## 2 Materials and Methods

### 2.1 Proteins and Chemicals

Recombinant human serum albumin, Albagen XL solution (UniProt ID: P02768) was purchased from Albumin Bioscience (Huntsville, AL, USA). The charcoal was purchased from Caesar & Loretz GmbH (Hilden, Germany). Hexafluoropropylene oxide-dimer acid (HFPO-DA or GenX) was purchased from SynQuest Laboratories (Alachua, FL, USA). C6O4 was purchased from Wellington laboratories (Ontario, Canada). Perfluorohexanoic acid (PFHxA), sodium myristate (Myr), and warfarin (War) were purchased from (Merck, Milan, Italy). Ibuprofen (Ibu) was purchased from Cayman Chemical (Ann Arbor, MI, USA). All the reagents were of analytical grade, and solutions were prepared using double distilled deionized water.

### 2.2 Protein Preparation and Purification

The defatted recombinant human serum albumin (dhSA) was obtained by adsorption onto activated charcoal as previously described.^46,47^ Briefly, the water-washed charcoal (0.4 mg per mg of hSA) was initially dissolved in PBS pH 7.4, and the pH was further lowered to 3 using a 1 M HCl solution. The resulting suspension was incubated for at least 3 hr. under gentle shaking at 4 °C. The pH of the suspension was then adjusted to 7.4 by using a 2 M NaOH solution and filtered using a 0.22 μm membrane filter. The protein aggregates and the disulfide-bridged dimers formed during this treatment were removed by size exclusion chromatography (SEC) using a HiLoad 16/600 Superdex 200 prep grade column (GE Healthcare, Milan, Italy) connected to an ÄKTA pure 25 M system (GE Healthcare, Milan, Italy) equilibrated with 50 mM sodium phosphate buffer (NaPi), 100 mM NaCl, pH 7.4. The fractions containing monomeric dhSA protein were pooled and further concentrated by using 10.000 NMWL Amicon Ultra-15 ultrafiltration devices (Merck, Milan, Italy) at 4000 g and 4 °C on a Heraeus Multifuge X1R centrifuge (Thermo Fisher Scientific, Waltham, MA, USA) to a final protein concentration of 25 mg/mL (375 μM). Protein concentration was determined using a mySPEC spectrophotometer (VWR, Radnor, PA, USA). Purified dhSA protein was flash-frozen in liquid nitrogen and stored at −80 °C. The monodisperse state of concentrated dhSA protein was confirmed by SEC using a Superdex 200 10/300 GL column (GE Healthcare, Milan, Italy) connected to an ÄKTA pure 25 M system and equilibrated with 50 mM NaPi, 100 mM NaCl, pH 7.4. Purified dhSA proteins were eluted as a single peak at elution volumes that corresponds to an apparent molecular mass of about 66 kDa (monomer).

### 2.3 Isothermal Titration Calorimetry

ITC experiments were performed using a Microcal PEAQ-ITC instrument (Malvern Panalytical, Malvern, UK). Both dhSA (110 μM) and ligands (HPFO-DA, C6O4, PFHxA, Ibu, and War, 4 mM solutions) were dissolved in 50 mM NaPi, 100 mM NaCl, pH 7.4. Only in the case of War, 2.5% v/v DMSO was added to the buffer to improve its solubility. All working solutions were properly degassed. Titrations of dhSA with ligands were carried out at different temperatures (298 and 310 K). In each experiment, an initial 0.4 μL injection (excluded from subsequent data analysis) was followed by 25 independent injections of 1.5 μL with a stirring rate of 750 rpm to ensure rapid mixing. A 120 s interval between injections was applied to guarantee the equilibrium at each titration point. Blank experiments (ligands against buffer) were carried out for each ligand and subtracted to corresponding titrations in order to screen dilution heat contributions. Competition experiments were conducted by adding saturating concentrations of albumin-binding drugs (either 250 μM Ibu or 200 μM War) to dhSA in the measure cell and titrating each PFAS (4 mM) with the aforementioned instrumental parameters. Data were analyzed using the MicroCal PEAQ-ITC Evaluation software (Malvern Panalytical, Malvern, UK). Integrated heat signals were fitted to “one set of sites” (C6O4, PFHxA), “two sets of sites” (PFOA), or “sequential” (HPFO-DA) binding models. Values for the binding affinity constant (*K*_A_ = 1/*K*_D_) and enthalpy change (Δ*H*), together with the stoichiometry of each PFAS-dhSA reaction, were obtained from curve fitting. Free energy and the entropy change (Δ*S*) were calculated from the Gibbs free energy (Δ*G*) relationships: Δ*G =* Δ*H* – *T*Δ*S =* −*RT* ln(*K*_A_).

### 2.4 Crystallization

Crystallization trials of dhSA in complex with HPFO-DA, C6O4 and sodium myristate (Myr) were carried out at 285 K in an MRC maxi 48-well crystallization plate (Hampton Research, Aliso Viejo, CA, USA) using the sitting-drop vapor-diffusion method and the Morpheus MD1–46 protein crystallization screen kit (Molecular Dimensions Ltd., Catcliffe, UK). Droplets of 1.6 μL volume (0.8 μL of protein complex and 0.8 μL of reservoir solution) were set up using an Oryx 8 crystallization robot (Douglas Instruments Ltd., Catcliffe, UK) and equilibrated against 120 μL reservoir solution. In all the cases, best crystals were obtained by streak- or micro-seeding and left for 5–7 days. Crystals of dhSA (1 mM) incubated with a 20-fold molar excess of HPFO-DA (20 mM) and a fivefold molar excess of Myr (5 mM) were obtained using the following precipitant agents: 50 mM bicine, 50 mM Trizma base, 30 mM sodium fluoride, 30 mM sodium bromide, 30 mM sodium iodide, 12.5% v/v MPD, 12.5% w/v PEG 1000, and 12.5% w/v PEG 3350 pH 8.5. For X-ray data collection, crystals were mounted on LithoLoops (Molecular Dimensions Ltd., Catcliffe, UK), soaked in cryoprotectant solution (crystallization buffer added with 20% v/v ethylene glycol), and flash-frozen in liquid nitrogen.

### 2.5 X-ray Diffraction Data Collection and Processing

X-ray diffraction data of the complexes were collected at ID23–2 beamline of the European Synchrotron Radiation Facility (ESRF, Grenoble, France). The best crystals of the ternary complex hSA-HPFO-DA-Myr (1:20:5 molar ratio) diffracted to 2.10 Å maximum resolution. Crystals belong to the C2 space group, with unit cell parameters: *a* = 185.89 Å, *b* = 38.77 Å, *c* = 96.45 Å, α = 90°, β = 105°, and γ = 90°. The asymmetric unit contains one molecule, corresponding to a Matthews coefficient of 2.45 Å^3^/Da and a solvent content of 49.84% of the crystal volume. Frames were indexed and integrated with software XIA2, merged, and scaled with AIMLESS (CCP4i2 crystallographic package).^48^

### 2.6 Structure Determination and Model Refinement

The structure was solved by molecular replacement with software PHASER^49^ using as a template the model 7AAI.^45^ Refinement was carried on using REFMAC^50^ and PHENIX.^51^ Rebuilding and fitting of the HPFO-DA, Myr, and precipitant/additive molecules (2-methyl-2,4-pentanediol, MPD; and Br) were performed manually with graphic software COOT.^52^ Since the first cycles of refinement, the electron density corresponding to the bound HPFO-DA and/or Myr molecules was clearly visible in the electron density map. The final model of the ternary complex hSA-HPFO-DA-Myr contains 4643 protein atoms, 40 HPFO-DA ligand atoms, 64 Myr ligand atoms, 34 water molecules, and 49 atoms of other molecules. The final crystallographic R factor is 0.237 (*R*_free_ 0.264). Geometrical parameters of the two models are as expected or better for this resolution. Intramolecular and intermolecular hydrogen bond interactions were analyzed by PROFUNC,^53^ LIGPLOT+,^54^ and PYMOL software.^55^ The Protein Data Bank (PDB) identification code for the hSA-HPFO-DA-Myr ternary complex is 7Z57.

## 3 Results and Discussion

### 3.1 Binding Kinetics of Branched Short-Chain HPFO-DA and C6O4 with hSA

**Figure 1:**
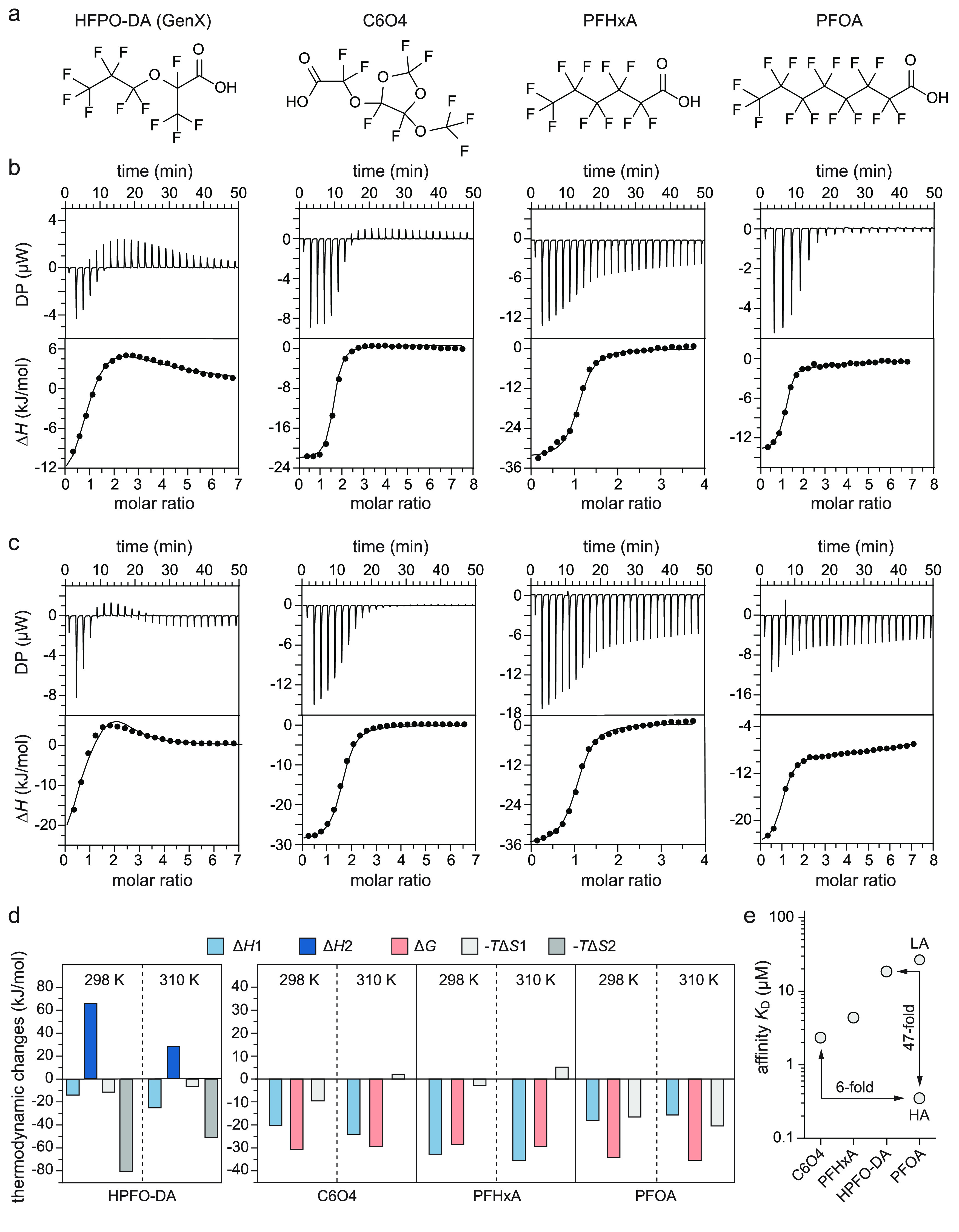
Binding kinetics of branched short-chain HPFO-DA and C6O4 to hSA compared to those of linear PFHxA (short-chain) and PFOA (long-chain) to hSA. (a) Chemical structure of HPFO-DA, C6O4, PFHxA, and PFOA; (b, c) ITC analysis of hSA binding to PFASs performed at two different temperatures. Representative raw trace (top) and integrated binding isotherm (bottom) of the calorimetric titration of HPFO-DA, C6O4, PFHxA, and PFOA at 298 K (b) and 310 K (c); (d) comparative analysis of the thermodynamic parameters: bar diagram representing the difference in enthalpy (Δ*H*1 and Δ*H*2, light and dark blue), in free Gibson energy (Δ*G*, light red) and entropy (−*T*Δ*S*1 and −*T*Δ*S*2, light and dark gray) for each dhSA-PFAS complex according to the fitting selected (sequential binding for HPFO-DA, one set of sites for C6O4 and PFHxA and two sets of sites for PFOA). For PFOA, only the parameters describing the high-affinity sites are reported; (e) summary of the *K*_D_ values (light gray) of HPFO-DA, C6O4, PFHxA, PFOA-HA (HA = high-affinity binding sites) and PFOA-LA (LA = low-affinity binding sites) at 298 K.

To determine the binding affinities of branched short-chain HPFO-DA and C6O4 to hSA, we used ITC. In-solution studies were performed at room (298 K) and physiological (310 K) temperatures. Linear long- and short-chain perfluoroalkyl compound PFOA and PFHxA, respectively, were included as controls (Figure 1a). Linear PFHxA was chosen because it contains the same number of perfluorinated carbons of both branched short-chain HFPO-DA and C6O4. PFOA was instead selected because it represents the prototype of perfluoropolyethers with a carboxylic head, and it is one of the most widely studied perfluoroalkyl substances. The binding mode of branched C6O4 to hSA resembled that of linear PFHxA (Figure 1b). Both short-chain PFASs appear to form stable complexes with hSA presenting similar binding affinity constants (C6O4: *K*_D_ = 2.4 ± 0.1 μM; PFHxA: *K*_D_ = 4.5 ± 2.1 μM; Figure 1b,c and Table 1). Binding isotherms at 298 and 310 K were best fitted with a “one set of sites” model revealing a 1:2 and 1:1 hSA:PFAS stoichiometry for C6O4 and PFHxA, respectively (Figure 1b,c and Table 1). Their binding kinetics are characterized by an exothermic reaction with favorable enthalpic (Δ*H*) and entropic (Δ*S*) contributions, suggesting that the formation of both hSA-C6O4 and hSA-PFHxA complexes is achieved through electrostatic and hydrophobic contacts (Figure 1d and Table 1). Inversely, binding of branched HPFO-DA to hSA was best fitted using a “sequential binding” model, with a first high-affinity binding event (*K*_D_ = 19 ± 1.3 μM) followed by a second lower affinity one (*K*_D_ > 80 μM; Figure 1b,c and Table 1). The initial binding event resembled that of the three low-affinity binding sites of PFOA (*K*_D_ = 29.7 ± 1.7 μM; Figure 1b,c and Table 1). The second binding event could instead be plausibly attributed to unspecific hydrophobic interactions occurring at high HPFO-DA concentrations. Indeed, this hypothesis is supported by the fact that the first binding event is enthalpically favored (−*T*Δ*S* = −12.9 ± 4.7; Δ*H* = −14.1 ± 4.9 kJ/mol), while the second binding event appears to be primarily entropically driven (Δ*H* = 33.8 kJ/mol; −*T*Δ*S* = −57.1 kJ/mol; Figure 1d and Table 1). Notably, no significant variations were observed in the stoichiometry nor in the binding affinities when ITC studies were performed at temperatures of 298 or 310 K (Figure 1b–d and Table 1). Overall, our in-solution binding studies revealed that, though some differences exist in the biding mechanisms and thermodynamic profiles of herein tested branched and linear short-chain PFASs, all three tested perfluorinated compounds have a weaker affinity for hSA than PFOA (Figure 1e and Table 1).

**Table 1:** Thermodynamic and Stoichiometric Data for the Binding of dhSA to HPFO-DA, C6O4, PFHxA at Different Temperatures (298 and 310 K)^a^

one set of sites				
	C6O4		PFHxA	
	298 K	310 K	298 K	310 K
*K*_D_ (μM)	2.4 ± 0.1	5.4 ± 2.1	4.5 ± 2.10	5.9 ± 0.70
Δ*H* (kJ/mol)	–21.4 ± 1.4	–25.8 ± 4.2	–34.1 ± 7.40	–39.2 ± 2.00
Δ*G* (kJ/mol)	–32.10	–31.70	–30.60	–31.70
–*T*Δ*S* (kJ/mol)	–10.7	1.7	–3.00	7.70
*n*	1.6 ± 0.2	1.5 ± 0.3	1.02 ± 0.05	1.03 ± 0.05

sequential binding		
	HPFO-DA	
	298 K	310 K
*K*_D1_ (μM)	19.0 ± 1.3	9.9 ± 2.3
Δ*H*_1_ (kJ/mol)	–14.1 ± 4.9	–23.2 ± 3.4
–*T*Δ*S*_1_ (kJ/mol)	–12.9	–6.54
*K*_D2_ (μM)	84.2 ± 14.6	27.0 ± 8.1
Δ*H*_2_ (kJ/mol)	33.8 ± 1.9	28.2 ± 2.8
–*T*Δ*S*_2_ (kJ/mol)	–57.1	–55.4

two sets of sites				
	PFOA high-affinity sites		PFOA low-affinity sites	
	298 K	310 K	298 K	310 K
*K*_D_ (μM)	0.4 ± 0.01	39.3 ± 0.4	29.7 ± 1.7	46.0 ± 2.00
Δ*H* (kJ/mol)	–20.1 ± 1.10	–16.5 ± 1.9	–20.1 ± 1.1	–2.26 ± 0.75
Δ*G* (kJ/mol)	–36.10	–38.10	–26.60	–29.10
–*T*Δ*S* (kJ/mol)	–17.70	–21.6	–28.2	–31.30
*n*	0.89 ± 0.30	0.95 ± 0.2	4.5 ± 1.2	9.5 ± 0.20

a *K*_D_, equilibrium dissociation constant; Δ*H*, enthalpy change; Δ*G*, Gibbs free energy; Δ*S*, entropy change; *T*, temperature; *n*, stoichiometry. Mean values and error of each parameter have been obtained from the fitting.

### 3.2 Competition Binding Experiments Using Drugs with Known hSA Site-Selectivity

**Figure 2:**
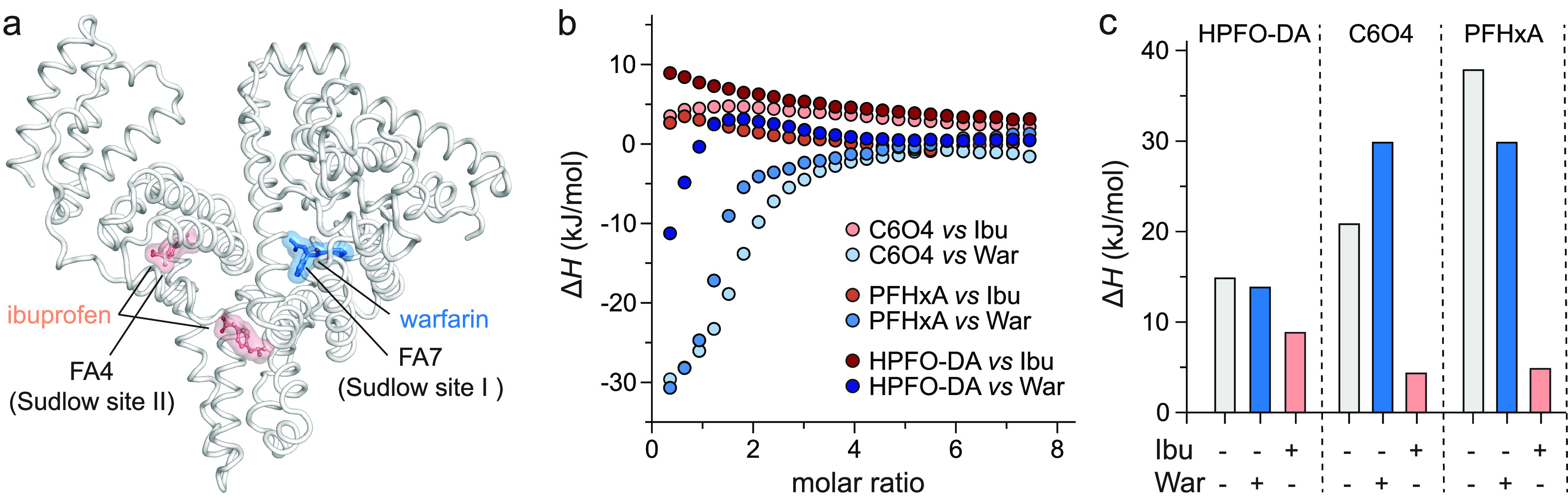
In-solution competitive binding experiments using drugs with known hSA site-selectivity. (a) Structural comparison of the ligand binding modes of ibuprofen (Ibu, salmon) and warfarin (War, sky blue) to hSA (white); (b) overlay of the individual titration profiles of short-chain HPFO-DA, C6O4, and PFHxA biding to hSA saturated with Ibu (red scale) or War (blue scale). The titration profiles are colored in light red and light blue for C6O4, red and blue for PFHxA, and dark red and dark blue for HPFO-DA; (c) difference in enthalpy (Δ*H*) of the HPFO-DA, C6O4, and PFHxA binding to hSA in absence of competitors (light gray), in the presence of ibuprofen (Ibu, salmon) or in the presence of warfarin (War, blue). The three-dimensional structure was generated and rendered using PYMOL.^55^

To assign the binding pockets of each branched short-chain PFAS, we performed competitive ITC studies using two commercially available drugs with known binding affinities and site-selectivity for hSA.^45^ These include ibuprofen (Ibu) and warfarin (War), two well-characterized molecules known to share the same fatty acid-binding sites (FA4 and FA6 for Ibu, FA7 for War) with long-chain PFASs (Figure 2a).^45^ Again, the short-chain perfluoroalkyl compound PFHxA was included as the control. Individual titration profiles of perfluorinated compounds HPFO-DA, C6O4, and PFHxA to hSA saturated with War were comparable to that of hSA in the absence of competitive drug, while those obtained in the presence of Ibu-saturated hSA revealed a nearly saturated flat curve, suggesting a direct competition of Ibu with both branched short-chain PFASs for FA4 and/or FA6 binding sites (Figures 2b and S1). The thermodynamics parameters of binding of both branched and linear tested short-chain PFASs to hSA in the presence of Ibu showed little or null enthalpic contribution, which was instead present when the hSA was saturated with War and comparable to that of hSA in the absence of competitive drugs (Figures 2b and S1). The results are consistent with those previously reported that identified the ibuprofen-binding pocket as a preferable cavity to lodge polyfluoroalkyl compounds.^45^ Given that all three tested perfluorinated compounds bind hSA with a 1:2 and 1:1 hSA:PFAS stoichiometry and appear to compete with Ibu, we can conclude that HPFO-DA, C6O4 and PFHxA preferably occupy FA4 and/or FA6 sites (Figure 2c).

### 3.3 Crystal Structure of hSA in Complex with HPFO-DA

**Figure 3:**
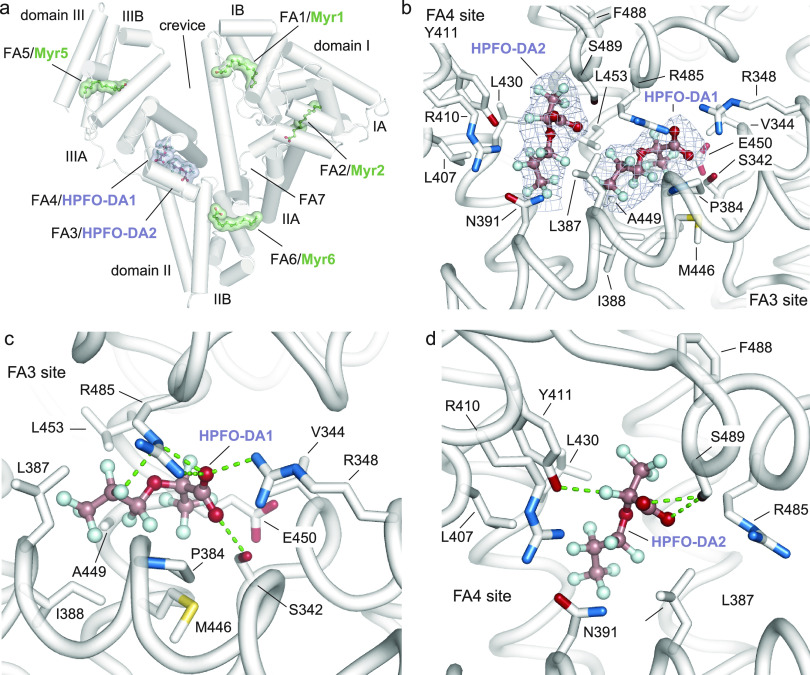
Crystal structure of branched short-chain HPFO-DA in complex with hSA. (a) Three-dimensional structure of hSA (white) in complex with two molecules of HPFO-DA (light purple) and Myr (green) [PDB identification code: 7Z57]. The α-helices of hSA are represented by cylinders. Bound HPFO-DA and Myr molecules are shown in a ball-and-stick representation with a semitransparent van der Waals (HPFO-DA: light purple, Myr: green) and colored by atom type (HPFO-DA: carbon = dark salmon, oxygen = firebrick, fluorine = pale cyan; Myr: carbon = smudge green, oxygen = firebrick); (b) HPFO-DA1 and HPFO-DA2 bound to FA3 and FA4 sites located in subdomain IIIA, respectively. Bound HPFO-DA molecules are depicted as ball-and-stick models and the composite omit maps, representing the (*F*_o_ – *F*_c_) electron density contoured at 4σ, are shown as light purple mesh.; (c) HPFO-DA1 bound to FA3 site; (d) HPFO-DA2 bound to FA4 site. The α-helices of hSA are shown in white and the selected amino acid side chains are represented as stick and colored by atom type (carbon = white, oxygen = firebrick, nitrogen = sky blue; sulfur = yellow orange). Hydrogen bonds, salt bridges, and polar interactions are shown as dashed lines. For visualization, only intermolecular polar interactions below 3.0 Å are shown. The three-dimensional structures were generated and rendered using PYMOL.^55^

To elucidate the binding mode of HPFO-DA and C6O4 to hSA, we applied X-ray crystallography and attempted to solve the three-dimensional structure of both perfluorinated compounds in complex with hSA. To better resemble the physiological conditions, cocrystallization trials were performed in the presence of a representative fatty acid, the myristic acid (Myr).^56^ Despite the numerous attempts, good-quality diffraction crystals were obtained only for hSA in complex with HPFO-DA and Myr. The best crystals diffracted to 2.2 Å maximum resolution, and the structure was solved by molecular replacement (Table S1, PDB identification code: 7Z57). A total of six distinct binding sites, two occupied by HPFO-DA and four by Myr, have been identified (Figure 3a). The electronic density of bound HPFO-DA ligands was clearly visible allowing a definite assignment of the positions and orientations of both hydrophilic carboxylate head groups and fluorinated lipophilic tails (Figure 3b). Both HPFO-DA molecules (herein named HPFO-DA1 and HPFO-DA2) occupy the long and narrow Sudlow’s drug-binding site II that span between FA3 and F4 sites, both located in subdomain IIIA (Figure 3a,b). The two molecules are closely located and positioned approximately at right angles to each other (Figure 3a,b). HPFO-DA1 is lying into the FA3 site while HPFO-DA2 lodges the F4 site (Figure 3b). The carboxylate head-group of HPFO-DA1 forms hydrogen bonds with the side chain of Ser342, Arg348, and Arg485 residues (Figure 3c). Furthermore, numerous polar interactions are established between fluorine atoms (F5, F6, F7, F14, F15, and F16) and the oxygen and nitrogen atoms of both main and side chains of nearby Ser342, Pro384, Ile388, Met446, Ala449, Glu450, and Arg485 residues (Figure 3c and Table S2). The rest of the fluorinated tail accommodates in the hydrophobic tunnel and establishes nonpolar contacts with surrounding Ser342, Val344, Arg348, Pro384, Leu387, Ile388, Met446, Ala449, Glu450, Leu453, and Arg485 residues (Figure 3c and Table S2). The hydrophilic carboxylate head-group of HPFO-DA2 is instead engaged in a hydrogen bond with the side chain of Ser489, while numerous polar interactions are established between fluorine atoms (F6, F12, F14, F15, F16, F18, and F19) and the oxygen and nitrogen atoms of both main and side chains of nearby Asn391, Tyr411, Leu430, Phe488, and Ser489 residues (Figure 3d and Table S2). The rest of the fluorinated tail accommodates in the hydrophobic tunnel and establishes nonpolar contacts with surrounding Leu387, Asn391, Leu407, Tyr411, Leu430, Arg485, Phe488, and Ser489 residues (Figure 3d and Table S2). Overall, binding of HPFO-DA molecules to FA3 and FA4 pockets involves both the carboxylate head-group and the fluorinated tail that establish polar and nonpolar contacts with surrounding hSA residues.

### 3.4 Differences in the Binding Mode of Branched Short-Chain HPFO-DA and Linear Long-Chain PFOA with hSA

**Figure 4:**
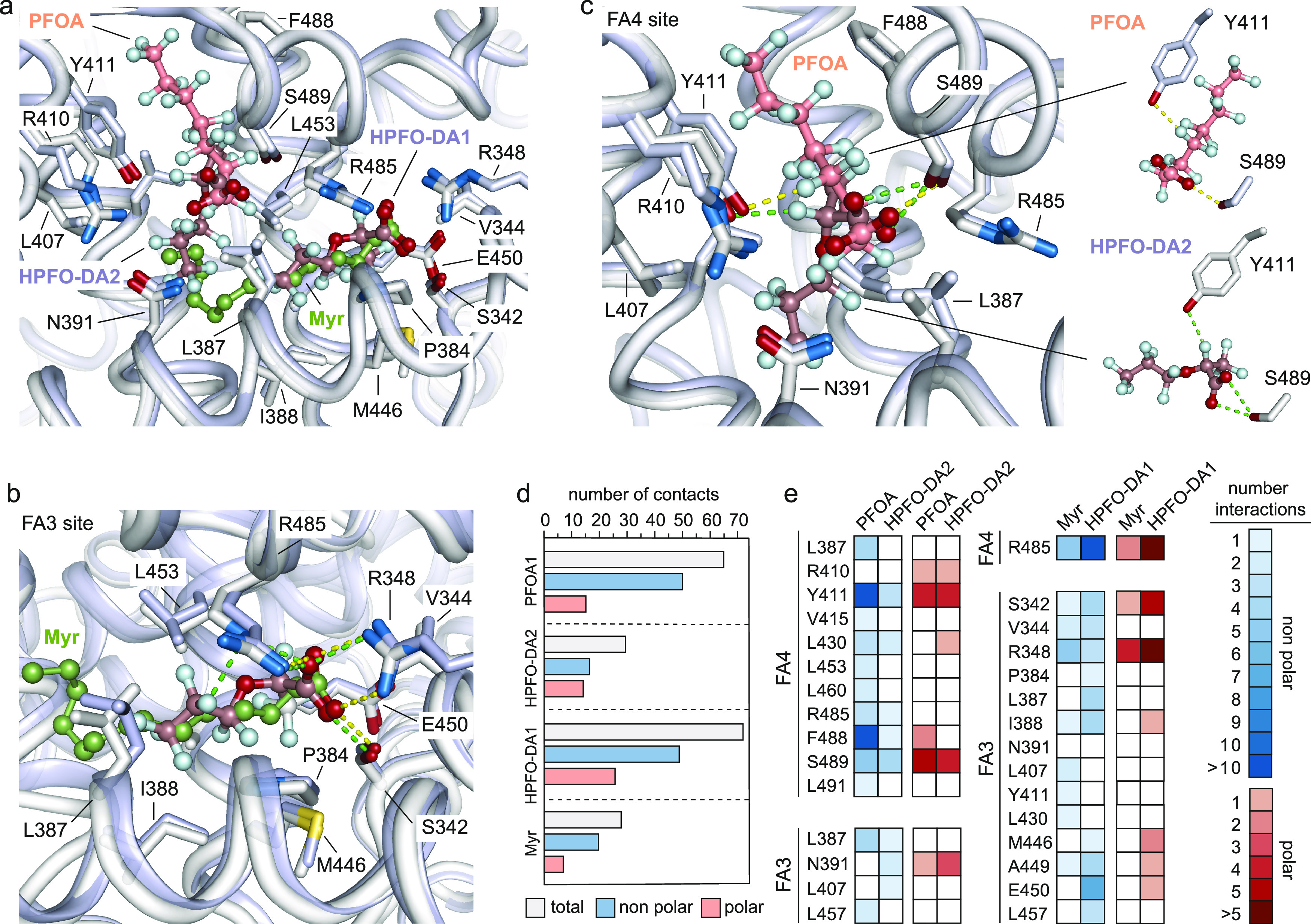
Structural comparison of the ligand binding modes of HPFO-DA and PFOA to hSA. (a) Detailed view of the superimposed PFOA, HPFO-DA1, HPFO-DA2, and Myr ligands bound to FA4 and FA3 sites in subdomain IIIA of hSA; (b) detailed view of the superimposed HPFO-DA1 and Myr ligands bound to the FA3 site; (c) detailed view of the superimposed HPFO-DA2 and PFOA ligands bound to the FA4 site. The α-helices of hSA in complex with PFOA and Myr are represented by light blue ribbon diagram while the α-helices of hSA in complex with HPFO-DA1 and HPFO-DA2 are represented by the white ribbon diagram. The selected amino acid side chains are represented as sticks and colored by the atom type (carbon = white for hSA-HPFO-DA-Myr complex and light blue for hSA-PFOA-Myr complex, oxygen = firebrick, nitrogen = sky blue, sulfur = yellow orange). Bound HPFO-DA, PFOA, Myr molecules are shown in a ball-and-stick representation and colored by the atom type (HPFO-DA: carbon = dirty violet, oxygen = firebrick, fluorine = pale cyan; PFOA: carbon = dark salmon, oxygen = firebrick, fluorine = pale cyan; Myr: carbon = smudge green, oxygen = firebrick). Only the side chains of amino acids of hSA forming intermolecular interactions below 4.0 Å are shown. Polar intermolecular interactions are represented as dashed lines. Those established by HPFO-DAs are colored in light green, while those formed by PFOA or Myr are colored in yellow; (d) columns graph reporting the number of total (light gray), nonpolar (light blue), and polar (light red) intermolecular interactions of hSA with HPFO-DA1, HPFO-DA2, PFOA, and Myr; (e) heat map visualization of the number of interactions of hSA residues involved in binding to HPFO-DA1 and Myr (left), HPFO-DA2 and PFOA (right). The residues of hSA are indicated as a three numbered letter code. Nonpolar interaction and polar interaction are shown in blue and red, respectively. The color intensity correlates with the number of the interactions, with numerous and few interactions shown as light and dark colors, respectively. The three-dimensional structures were generated and rendered using PYMOL.^55^

We next compared our hSA-HPFO-DA-Myr structure with that of hSA in complex with linear long-chain PFOA molecules (PDB identification code: 7AAI).^41,45^ The superposition of the two crystal structure complexes did not show any striking rearrangements of the main backbone with root mean square deviations over Cα-atoms of 0.48 Å (Figure S2). Though hSA amino acid side chains involved in the binding to HPFO-DA1 and HPFO-DA2 superimposed well with those of the hSA-PFOA-Myr complex, some differences appear to exist in the positioning of ligands inside the pockets (Figure 4a). Indeed, while the hSA-HPFO-DA-Myr complex revealed the presence of two HPFO-DA molecules bound to FA3 (HPFO-DA1) and F4 (HPFO-DA2) sites, respectively, the hSA-PFOA-Myr complex identified a single PFOA molecule located in the FA4 site and a Myr one into the FA3 pocket (Figure 4a). Both HPFO-DA1 and Myr in FA3 are positioned approximately at right angles to HPFO-DA2 and PFOA molecules located in the nearby FA4 site, respectively (Figure 4a). The hydrophilic carboxylate head-group and the lipophilic tail of HPFO-DA1 in FA3 superimposed well with that of Myr (Figure 4b). Both HPFO-DA1 and Myr ligands establish polar and nonpolar contacts with similar surrounding main and side chain hSA residues (Figure 4b). Most of the hSA side chains that are engaged in HPFO-DA1 binding displayed similar conformations of the hSA-PFOA-Myr complex except for a slight rotation of the Leu387 side chain (Figure 4b). Contrariwise, the binding mode of HPFO-DA2 and PFOA ligands to the FA4 site varies significantly (Figure 4b). While the hydrophilic head groups converge toward a common central polar residue (Ser489), with whom they establish a polar interaction, the fluorinated tails run in opposite directions exploiting different hSA residues (Figure 4c). Notably, F19 and F12 atoms of HPFO-DA1 and PFOA, respectively, establish similar fluorine polar interaction with Tyr411 (Figure 4c). Again, no major differences are observed for the position of side chains of hSA amino acids involved in contacts with HPFO-DA2 when compared to the hSA-PFOA complex (Figure 4c). Interestingly, molecular analysis of the intermolecular interactions established by HPFO-DA1 and HPFO-DA2 with surrounding hSA residues of FA3 and F4 pockets, respectively, revealed that HPFO-DA1 forms a larger number of polar and nonpolar contacts if compared to HPFO-DA2 (Figure 4d,e). Overall, we conclude that HPFO-DA1 appears to bind to FA3 similarly to Myr, while HPFO-DA2 occupies the FA4 site by exploiting different locations and hSA residues.

## 4 Discussion and Conclusions

Exposure of general population to PFASs occurs, in most cases, through oral ingestion of contaminated food or drinking water. After absorption in the gut, PFASs reach the bloodstream and distribute throughout the body, accumulating in certain organs or tissues. This latter event ultimately associates with adverse health outcomes. While the interaction of hSA with linear long-chain PFASs, such as PFOA and PFOS, has been already characterized using multiple methodologies, a detailed analysis of the binding mode of new-generation short-chain polyfluoroalkyl compounds to hSA is still lacking. Here, we report the biochemical characterization of hSA in complex with representative branched short-chain fluorinated compounds HPFO-DA and C6O4. In-solution ITC binding studies revealed the presence of one to two hSA binding sites for both branched short-chain PFASs. Our data are partially in agreement with those reported in previous studies describing the ability of hSA to bind several HPFO-DA molecules.^40,43^ The nature of the binding of HPFO-DA and C6O4 to hSA appears to be similar to that of linear short-chain (PFHxA) and long-chain (PFOA) compounds. Binding energetics appear to be driven by an exothermic process and by a gain in entropy most probably due to desolvation of the fluoroalkyl tail. The approximately 6- to 47-fold lower binding affinities determined for short-chain PFASs (C6O4, *K*_D_ = 2.4 μM; HPFO-DA, *K*_D_ = 19 μM), compared to that of their long-chain counterparts (*K*_D_ = 0.4 μM), can be mainly ascribed to their shorter hydrophobic tail that ultimately limit the number of hydrophobic interactions that can be established with the surrounding hSA residues. However, despite these major structural changes, the protein binding affinity of such short-chain PFAS to hSA should be elucidated case by case.^43,57^ In particular, given the similar size, we can speculate that the higher binding affinity measured for the C6O4 compound with respect to HPFO-DA and PFHxA could be related to the greater number of oxygen atoms present. Indeed, while HPFO-DA and PFHxA include only two and three oxygens, respectively, C6O4 contains six oxygens that can potentially function as hydrogen bond donors.^28^ However, this does not apply to HPFO-DA whose affinity for hSA is weaker than that of PFHxA, despite the presence of an additional oxygen atom. This suggests that the total number of atoms in the backbone might also play a role and that the intermolecular forces at stake are not easy to foresee. Further competition experiments with known hSA-binding drugs identified the Sudlow’s drug-binding site II in subdomain IIIA as the high-affinity binding site. The ability of all tested short-chain perfluorinated compounds to outcompete with Ibu for Sudlow’s drug-binding site II, a primary site for numerous drugs, is remarkable and supports the need to further investigate the resulting role in PFASs’ toxico-kinetics at different levels: from the impact on the serum half-life of the compound, largely ascribed to renal elimination of the “free” form unbound to hSA,^58^ to the interference with absorption and accumulation of other exogenous drugs and the related potential toxicity. Indeed, binding of PFASs to hSA, the major drug-carrier protein in blood plasma,^59^ might prevent or displace drug binding thus increasing concentration of the unbound drug in circulation, altering its pharmacological effect, and posing health risks. To unveil the molecular basis of the binding mode of both perfluorinated compounds to hSA we attempted to solve the three-dimensional structure of HPFO-DA-hSA and C6O4-hSA complexes. However, while good-quality diffraction crystals were readily attained for hSA in complex with HPFO-DA, no crystals were detected for hSA bound to C6O4 despite the numerous crystallization trials performed. Overall, the crystal structure of hSA in complex with HPFO-DA and Myr revealed the presence of two distinct HPFO-DA binding sites and no conformational differences with those of other hSA-FA complexes. Further comparison of the crystal structure of hSA in complex with HPFO-DA and Myr with that of hSA in complex with PFOA and Myr enabled us to appreciate the analogies and differences in their respective interaction mechanisms. For instance, our structural studies showed that HPFO-DA2 can access a different compartment of FA4 and explore novel hSA amino acids located at the interface between FA3 and FA4 sites otherwise inaccessible. This appears to depend on the presence of the smaller ligand HPFO-DA1 instead of the longer-chain Myr in the FA3 pocket. Indeed, the presence of a sterically bulky Myr that occupies entirely the FA3 pocket of the hSA-PFOA-Myr complex seems to prevent PFOA molecules from entering further deeply center of the pocket leaving it to sit in the middle of FA4 site. Again, our observations partially deviate from those reported in previous studies.^40,42,43,57^ While in-solution and structural molecular analysis here presented confirmed the presence of a HPFO-DA molecule (HPFO-DA2) bound to the FA4 site, no HPFO-DA ligand has been shown to bind to the FA7 site (Sudlow’s site I). Moreover, contrarily from what was anticipated from computational studies,^44^ HPFO-DA molecule positions differently into the FA4 site, and it does not engage in hydrogen bonds with nearby Arg410. Notably, our crystallographic studies unveiled the presence of an unpredicted HPFO-DA molecule (HPFO-DA1) bound to FA3. The ability of the short-chain HPFO-DA molecules to outcompete Myr not only for FA4 but also for FA3 site binding is surprising, especially in light of the fact that neither the long-chain PFOA was found to be able to displace Myr bound to FA3. Indeed, comparative analysis of the interactions of HPFO-DA ligands to the residues of hSA showed that the HPFO-DA1 molecule bound to FA3 establishes a higher number of intermolecular contacts if compared to the HPFO-DA2 molecule bound to FA4. We can speculate that the smaller size of HPFO-DA, with respect to PFOA, might have facilitated its diffusion into the Sudlow’s binding sites II (FA3–FA4), a long and narrow hydrophobic tunnel, known to discriminate ligands based on their size. One molecule of HPFO-DA could probably initially penetrate and lodge into the FA3 site followed by a second molecule of HPFO-DA that is instead located into the contiguous FA4 pocket. The simultaneous presence of two molecules of HPFO-DA into the FA3 and FA4 sites might induce a cooperative binding that ultimately contributes to displace the Myr3 situated in FA3. In conclusion, we have demonstrated that new-generation branched short-chain perfluoroalkyl compounds can bind hSA, though with weaker affinity as compared to the long-chain ones. Compared with legacy PFASs, these data support a lower affinity of new-generation PFAS alternatives to hSA, possibly suggesting a lower half-life in vivo, as already reported for HPFO-DA in rodents.^60^ However, the global health impact should necessarily consider the specific tissue and cell toxicity that in the case of C6O4 is still under investigation while for HPFO-DA is particularly relevant.^61^ Further toxicological and epidemiological studies are needed to address the complete toxicokinetic profile of new-generation alternatives to legacy PFAS.

## Supporting Information Available

Supplementary tables and supplementary figures, statistics on X-ray diffraction data collection and refinement, atoms of HPFO-DA forming intermolecular polar and nonpolar interactions with atoms and residues of hSA, isothermal titration calorimetry analysis of defatted hSA to HPFO-DA, C6O4, and PFHxA in the presence of hSA-binding drugs ibuprofen or warfarin, and superimposition of hSA-HPFO-DA-Myr and hSA-PFOA-Myr complexes (PDF)
