# Supplementary material for: Investigation of the Interaction between Human Serum Albumin and Branched Short-Chain Perfluoroalkyl Compounds
Source: Chem Res Toxicol. 2022 Sep 23;35(11):2049–58. doi: 10.1021/acs.chemrestox.2c00211 (PMC9682524; doi:10.1021/acs.chemrestox.2c00211)
Supplement: Supplementary file 1 — tx2c00211_si_001.pdf [file tx2c00211_si_001.pdf]

Supporting Information

Investigation of the interaction between human serum albumin and branched short-chain perfluoroalkyl compounds

Giulia Moro[1,3], Stefano Liberi[2,†], Filippo Vascon[2], Sara Linciano[1], Sofia De Felice[2], Silvano Fasolato[3], Carlo Foresta[4], Luca De Toni[4], Andrea Di Nisio[4], Laura Cendron[2], Alessandro Angelini[1,5*]*

[1] Department of Molecular Sciences and Nanosystems, Ca' Foscari University of Venice, Via Torino 155, 30172 Venice, Italy

[2] Department of Biology, University of Padua, Viale G. Colombo 3, 35131 Padua, Italy

[3] Department of Medicine, University of Padua, Via Giustiniani 2, 35128 Padova, Italy

[4] Department of Medicine, Unit of Andrology and Reproductive Medicine, University of Padua, Via Giustiniani 2, 35128 Padua, Italy

[5] European Centre for Living Technology (ECLT), Ca' Bottacin, Dorsoduro 3911, Calle Crosera, 30123 Venice, Italy

* Authors to whom correspondence should be addressed: laura.cendron@unipd.it and alessandro.angelini@unive.it

† Present address: The Armenise-Harvard Laboratory of Structural Biology, Department of Biology and Biotechnology "L. Spallanzani", University of Pavia, Via Ferrata 9, 27100 Pavia, Italy.

Table of contents:

Page S3 – S4 Supplementary table 1. Statistics on X-ray diffraction data collection and refinement.

Page S5 – S10 Supplementary table 2. Atoms of HPFO-DA forming inter-molecular polar and non-polar interactions with atoms and residues of hSA.

Page S11 Supplementary figure 1. Isothermal titration calorimetry analysis of defatted hSA to HPFO-DA, C6O4 and PFHxA in the presence of hSA-binding drugs ibuprofen (Ibu) or warfarin (War) at 298 K.

Page S12 Supplementary figure 2. Superimposition of hSA-HPFO-DA-Myr [PDB ID: 7Z57] and hSA-PFOA-Myr [PDB ID: 7AAI] complexes.

Page S13 References

Table S1. Statistics on X-ray diffraction data collection and refinement. A single crystal was used to collect all diffraction data. Highest-resolution shell statistics are shown within brackets.

**Data collection ***	**hSA-HPFO-DA-Myr**
Beamline	ID 23-2
Wavelength (Å)	0.8731
Space group	C 1 2 1
Cell parameters	
a, b, c (Å); α, β, γ (°)	185.89 38.77 96.45 90 105.0 90
Resolution (Å)	93.16 – 2.2 (2.28 – 2.2)
Unique observations	34368 (3431)
Multiplicity	5.5 (5.6)
R_{merge}	0.079 (0.78)
R_{pim}	0.036 (0.219)
<I / σ(I)>	14.3 (2.27)
CC1/2	0.998 (0.81)
Completeness (%)	99.70 (99.77)
**Refinement**	
No. reflections (used for R_{free} calculation)	34341 (1715)
R_{work} / R_{free}	0.237 / 0.259

Number non-hydrogen atoms	4830
protein (chain A)	4643
ligands (HPFO-DA, Myr, Br)	153
solvent	34
**Geometry**	
RMSD values	
bond lengths (Å)	0.013
bond angles (°)	1.55
Ramachandran plot (%)	
most favored	97.06
additionally allowed	2.76
outliers	0.17
Average B-factor	50.66

Table S2. Atoms of HPFO-DA forming inter-molecular polar and non-polar interactions with atoms and residues of hSA (PDB ID: 7Z57). Optimal inter-molecular hydrogen bonds (HB) and polar interactions (PI) were defined using PROFUNC [1] and LIGPLOT+ [2]. Not specified interactions are non-polar. Interactions have distances shorter than 4.0 Å.

Binding site	hSA atom / residue	atom, interaction, distance (Å)	
		HPFOA-DA1	HPFOA-DA2
FA4	NH1 / Arg410	F18 (PI, 3.68)	
	OH / Tyr411	F19 (HB, 2.54)	
	OH / Tyr411	C03 (3.73)	
	OH / Tyr411	F15 (PI, 3.86)	
	OH / Tyr411	F16 (PI, 3.42)	
	OH / Tyr411	F19 (PI, 2.54)	
	CE1 / Tyr411	F19 (3.45)	
	CZ / Tyr411	F19 (3.35)	
	O / Leu430	F14 (PI, 3.85)	
	CB / Leu430	F15 (3.71)	
	CD2 / Leu430	F15 (3.60)	
	NE / Arg485		O20 (HB, 2.85)
	NH2 / Arg485		O20 (HB, 2.87)
	CD / Arg485	O20 (3.33)	

	CB / Arg485		F19 (3.83)
	CB / Arg485		O20 (3.89)
	CG / Arg485		F15 (3.48)
	CG / Arg485		O08 (3.79)
	CD / Arg485		C10 (3.70)
	CD / Arg485		F15 (3.08)
	CD / Arg485		F16 (3.31)
	CD / Arg485		O08 (3.72)
	NE / Arg485		C01 (3.64)
	NE / Arg485		C03 (3.85)
	NE / Arg485		C10 (3.59)
	NE / Arg485		F15 (PI, 3.47)
	NE / Arg485		F16 (PI, 2.88)
	NE / Arg485		O08 (PI, 3.32)
	NE / Arg485		O20 (PI, 2.85)
	CZ / Arg485		F16 (3.03)
	CZ / Arg485		O20 (3.27)
	NH1 / Arg485		F16 (PI, 3.52)
	NH2 / Arg485		C01 (3.76)
	NH2 / Arg485		F16 (PI, 3.48)

	NH2 / Arg485		O20 (PI, 2.87)
	CB / Phe488	F06 (3.85)	
	N / Ser489	F06 (PI, 3.85)	
	CA / Ser489	O02 (3.71)	
	CB / Ser489	O02 (3.50)	
	CB /Ser489	O20 (3.88)	
	OG / Ser489	C01 (3.02)	
	OG / Ser489	F06 (PI, 3.39)	
	OG / Ser489	O02 (PI, 2.96)	
	OG / Ser489	O20 (PI, 2.67)	
**FA3**	OG / Ser342		F06 (HB, 3.34)
	OG / Ser342		O02 (HB, 2.91)
	CB / Ser342		C04 (3.75)
	CB / Ser342		F06 (3.21)
	CB / Ser342		F07 (3.25)
	CB / Ser342		O02 (3.73)
	OG / Ser342		F06 (PI, 3.34)
	OG / Ser342		F07 (PI, 3.55)
	OG / Ser342		O02 (PI, 2.91)
	CG2 / Val344		C01 (3.76)

	CG2 / Val344		F07 (3.06)
	CG2 / Val344		O02 (3.40)
	NH1 / Arg348		O20 (HB, 3.33)
	NH2 / Arg348		O20 (HB, 2.62)
	CZ / Arg348		O20 (3.39)
	NH1 / Arg348		C01 (3.80)
	NH1 / Arg348		O02 (PI, 3.35)
	NH1 / Arg348		O20 (PI, 3.33)
	NH2 / Arg348		C01 (3.48)
	NH2 / Arg348		O02 (PI, 3.71)
	NH2 / Arg348		O20 (PI, 2.62)
	O / Pro384		F14 (PI, 3.14)
	CB / Pro384		F17 (3.40)
	CB / Leu387		F13 (3.51)
	CG / Leu387		F13 (3.35)
	CD2 / Leu387	O20 (3.47)	F13 (3.34)
	CD2 / Leu387		F16 (3.55)
	N / Ile388		F14 (PI, 3.84)
	CG1 / Ile388		C11 (3.72)
	CG1 / Ile388		F12 (3.82)

	CG1 / Ile388		F14 (2.79)
	CD1 / Ile388		F14 (3.23)
	CB / Asn391	F12 (3.53)	
	CG / Asn391	F12 (3.71)	
	OD1 / Asn391	F16 (PI, 3.84)	
	ND2 / Asn391	F12 (PI, 3.87)	
	ND2 / Asn391	F18 (PI, 3.85)	
	CD2 / Leu407	F16 (3.67)	
	O / Met446		F05 (PI, 3.80)
	O / Met446		F06 (PI, 3.68)
	CG / Met446		F06 (3.01)
	C / Ala449		F05 (3.43)
	O / Ala449		F05 (PI, 3.89)
	CB / Ala449		F05 (3.65)
	CB / Ala449		F12 (3.61)
	CB / Ala449		F18 (3.26)
	N / Glu450		F05 (PI, 2.99)
	CA / Glu450		F05 (3.10)
	CA / Glu450		F07 (3.69)
	CB / Glu450		C04 (3.78)

	CB / Glu450		F05 (3.45)
	CB / Glu450		F07 (3.19)
	CG / Glu450		F07 (3.46)
	CD / Glu450		F07 (3.78)
	CD1 / Leu453		F05 (3.87)
	CD1 / Leu453		F15 (3.18)
	CD1 / Leu453		O08 (3.83)

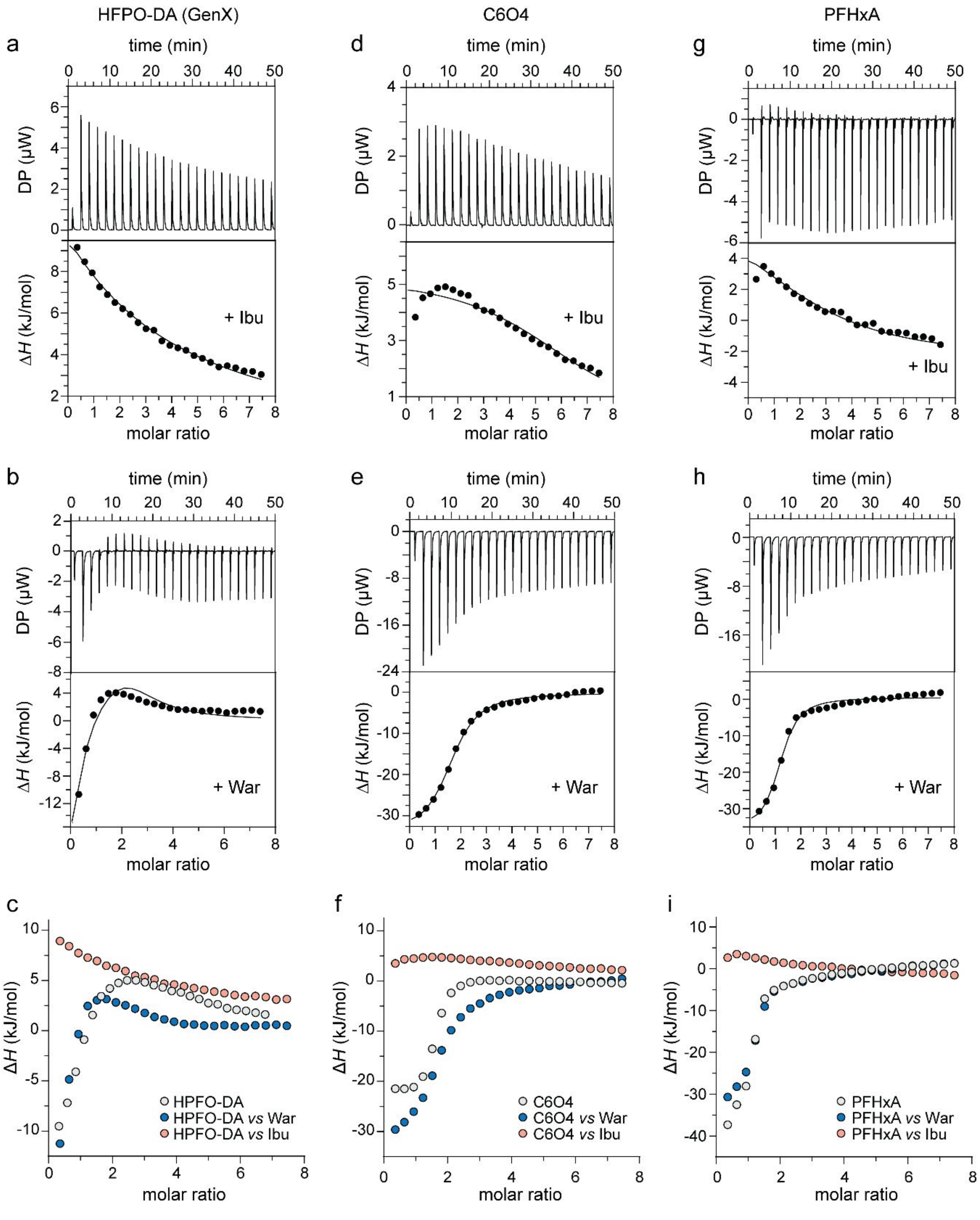

Figure S1. Isothermal titration calorimetry analysis of defatted hSA to HPFO-DA, C6O4 and PFHxA in the presence of hSA-binding drugs ibuprofen (Ibu) or warfarin (War) at 298 K. Representative raw trace (top) and integrated binding isotherm (bottom) of the calorimetric titration of HPFO-DA (**a-b**), C6O4 (**d-e**) and PFHxA (**g-h**). Single titration profiles of C6O4

(**c**), PFHxA (**f**) and HPFO-DA (**i**) in absence of competitors (light grey), in the presence of ibuprofen (Ibu, light red) or in the presence of warfarin (War, light blue).

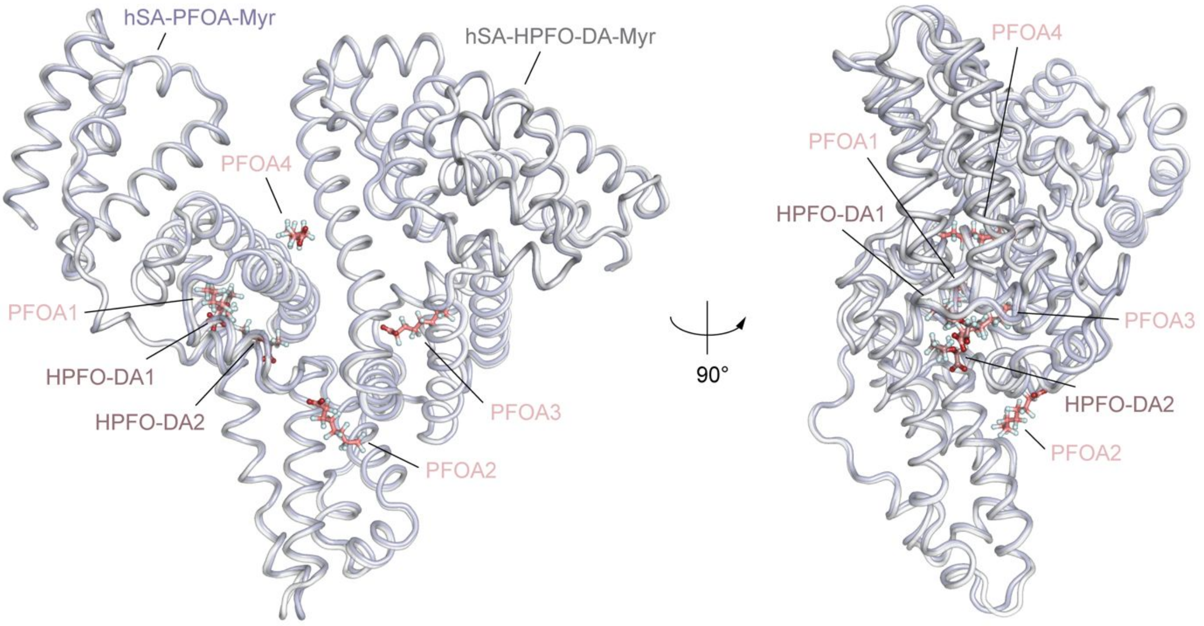

Figure S2. Superimposition of hSA-HPFO-DA-Myr [PDB ID: 7Z57] and hSA-PFOA-Myr [PDB ID: 7AAI] complexes. The overlaid α-helices of hSA-HPFO-DA-Myr (white, dirty violet) and hSA-PFOA-Myr (light blue, dark salmon) complexes are shown by ribbon diagrams and in two orientations (90˚ rotation). The three-dimensional superimposed structures were generated and rendered using PYMOL [3].

References

(1) Laskowski, R. A.; Watson, J. D.; Thornton, J. M. ProFunc: A Server for Predicting Protein Function from 3D Structure. *Nucleic Acids Res.* **2005**, *33* (suppl_2), W89–W93. https://doi.org/10.1093/nar/gki414.

(2) Laskowski, R. A.; Swindells, M. B. LigPlot+: Multiple Ligand-Protein Interaction Diagrams for Drug Discovery. *J. Chem. Inf. Model.* **2011**, *51*, 2778–2786. https://doi.org/10.1021/ci200227u.

(3) The PyMOL Molecular Graphics System, Version 2.0 Schrödinger, LLC.
